# Relationships between Electroencephalographic Spectral Peaks Across Frequency Bands

**DOI:** 10.3389/fnhum.2013.00056

**Published:** 2013-03-04

**Authors:** S. J. van Albada, P. A. Robinson

**Affiliations:** ^1^Institute of Neuroscience and Medicine (INM-6) and Institute for Advanced Simulation (IAS-6), Jülich Research Centre and Jülich-Aachen Research AllianceJülich, Germany; ^2^School of Physics, The University of SydneySydney, NSW, Australia; ^3^Brain Dynamics Center, Sydney Medical School – Western, University of SydneySydney, NSW, Australia; ^4^Center for Integrated Research and Understanding of SleepGlebe, NSW, Australia

**Keywords:** EEG, spectra, peaks, mean-field model, neural field theory, harmonic frequencies

## Abstract

The degree to which electroencephalographic spectral peaks are independent, and the relationships between their frequencies have been debated. A novel fitting method was used to determine peak parameters in the range 2–35 Hz from a large sample of eyes-closed spectra, and their interrelationships were investigated. Findings were compared with a mean-field model of thalamocortical activity, which predicts near-harmonic relationships between peaks. The subject set consisted of 1424 healthy subjects from the Brain Resource International Database. Peaks in the theta range occurred on average near half the alpha peak frequency, while peaks in the beta range tended to occur near twice and three times the alpha peak frequency on an individual-subject basis. Moreover, for the majority of subjects, alpha peak frequencies were significantly positively correlated with frequencies of peaks in the theta and low and high beta ranges. Such a harmonic progression agrees semiquantitatively with theoretical predictions from the mean-field model. These findings indicate a common or analogous source for different rhythms, and help to define appropriate individual frequency bands for peak identification.

## 1 Introduction

Electroencephalographic (EEG) spectra are often characterized by peaks at various frequencies. Most notable is the alpha peak, which usually lies between 8 and 12 Hz in healthy adult humans. It was the first feature reliably detected in human EEG (Berger, 1933), and has often been subcategorized into variants in different regions of the cortex (Niedermeyer and Lopes da Silva, 2005). Other peaks have been widely noted, including beta peaks typically in the range 13–30 Hz in healthy adults, spatially localized gamma peaks above 30 Hz, the theta peak at 4–8 Hz, and (in sleep) spindle peaks at 11–15 Hz (Niedermeyer and Lopes da Silva, 2005). All these peaks are superposed on broadband activity that falls off with increasing frequency.

**Figure 1:**
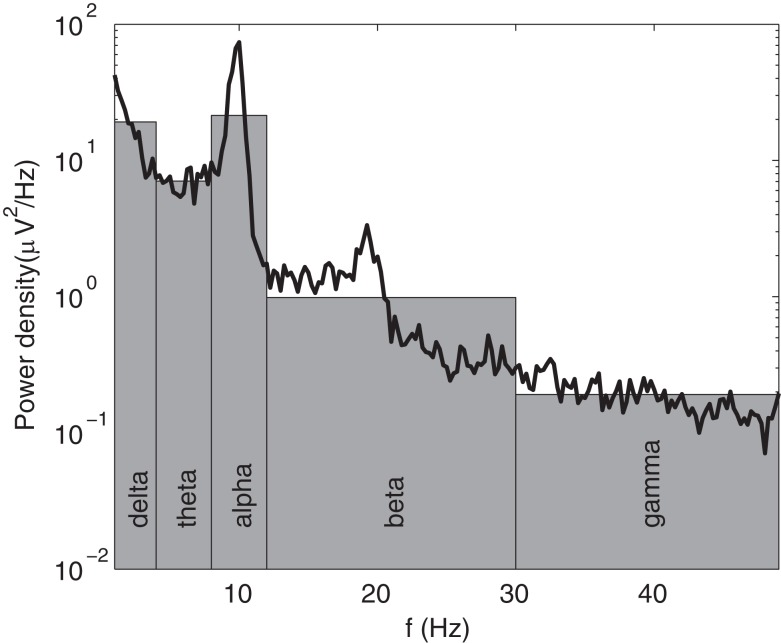
**Example of an EEG spectrum (black line) with its qEEG approximation in terms of band powers, given by the areas of the gray bars**.

In the most common forms of quantitative EEG (qEEG), the frequency spectrum is divided into several bands, and the total absolute or relative power in each band is analyzed. While the analysis of band powers has proved to be useful, it amounts to approximating the rich structure of actual EEG spectra by just a few numbers (see Figure 1). Moreover, the bands used to calculate these powers are almost invariably based on average parameters for normal adult humans. This procedure for instance fails to capture the fact that the alpha peak rises from around 3–5 Hz in newborns (Niedermeyer, 1997; Marshall et al., 2002) to 7–13 Hz in normal adults (Van Albada et al., 2010; Chiang et al., 2011), and individual variability which can take peaks outside the normal ranges. In the present study, we perform EEG spectroscopy of a large sample of healthy individuals, characterizing spectral structure in detail, and allowing for individual variations in frequency bands.

Various EEG rhythms have been noted to reflect different states of vigilance or independent aspects of cognitive processing (Niedermeyer and Lopes da Silva, 2005). For example, the alpha peak is most prominent in the eyes-closed condition and is associated with attentional suppression (Snyder and Foxe, 2010), while a spindle peak is associated with non-REM sleep, and theta peaks occur especially during drowsiness (Niedermeyer and Lopes da Silva, 2005). Characterizing the relationships between spectral peaks helps to refine such interpretations and sheds light on the underlying mechanisms.

Multiple suggestions have been made as to why EEG peaks have the observed frequencies:

(i) Based on spectral estimates in rats, it was suggested that successive functional frequency bands increase in center frequency by a factor *e* ≈ 2.718 (Penttonen and Buzsáki, 2003; Buzsáki and Draguhn, 2004). In rat brain slices, oscillations could be induced at relative frequencies corresponding approximately to the golden ratio, suggesting period concatenation as an underlying mechanism (Roopun et al., 2008a,b). Since the golden ratio is close to *e*^0.5^, the second proposal is related to the first, but implies a denser packing of rhythms across frequency. Both Euler’s number and the golden ratio were proposed by offer a computational advantage by minimizing interference between rhythms (Roopun et al., 2008a,b; Pletzer et al., 2010).
(ii) A second suggestion is that rhythms are produced by groups of neurons with similar characteristic frequencies, which might synchronize and act as “pacemakers.” Despite the existence of neurons with intrinsic oscillation properties, this hypothesis suffers from a number of drawbacks (Nunez and Srinivasan, 1981); for instance, it would require a separate pacemaker to be postulated *ad hoc* for each spectral peak.
(iii) Nunez suggested that global EEG rhythms arise as spatial cortical eigen-modes, yielding a non-harmonic progression of peak frequencies (Nunez and Srinivasan, 1981; Nunez, 1995). One prediction of this hypothesis is that alpha frequency should be negatively related to head size, which was found by Nunez (1978) and Posthuma et al. (2001) but was recently challenged (Valdés-Hernández et al., 2010).
(iv) Several other models have considered purely cortical oscillations (Van Rotterdam and Lopes da Silva, 1982; Liley et al., 1999, 2002; Wright, 1999; Jirsa et al., 2002; David and Friston, 2003). For instance, networks of simulated multicompartmental cortical neurons can produce oscillations in the range 8–20 Hz (Liley et al., 1999), and in a non-linear continuum theory, peaks at various frequencies in the range 2–16 Hz were obtained depending on the parameters (Liley et al., 2002).
(v) Considerations of the importance of the thalamus in synchronized oscillations in both sleeping and waking states (Lopes da Silva et al., 1973, 1980; Steriade et al., 1993, 1996; Steriade, 2000) have motivated thalamocortical models (Lumer et al., 1997; Robinson et al., 2001b, 2002; Rennie and Robinson, 2002; Hill and Tononi, 2005; Izhikevich and Edelman, 2008). The proposed models display resonances in various ranges: Lumer et al. (1997) found mostly gamma oscillations with precise frequencies depending on the parameters, Izhikevich and Edelman (2008) found oscillations in the delta and alpha ranges, and the model of Hill and Tononi (2005) exhibited slow waves in sleep and gamma oscillations in activated states. The neural field models of Rennie and Robinson (2002) and Robinson et al. (2001b, 2002), which are further explored here, provide a unified mechanism for slow-wave and spindle oscillations in sleep, and alpha, beta, and higher-frequency oscillations in the waking state. These models predict clear relationships between peak frequencies and amplitudes, with the theta peak occurring at approximately half the alpha frequency on an individual-subject basis, and alpha and beta peaks forming part of a near-harmonic progression.

The latter prediction is consistent with a number of previous studies: Carlqvist et al. (2005) found clear frequency, power, and phase relationships between alpha and beta activity in the resting EEG. The average ratio between beta and alpha peak frequencies was 1.9–2.0, consistent with the beta peak being generated as a harmonic of alpha. Similarly, bispectral analysis of subjects with high alpha activity revealed significant phase and amplitude relationships between alpha and its second harmonic (Barnett et al., 1971). In addition, Barnett et al. (1971) observed that 10 Hz activity was significantly phase-related to third and fourth harmonics at 30 and 40 Hz in some cases, and less prominently to activity at 2 and 7 Hz. Palva et al. (2005) reported cross-frequency phase synchrony between alpha, beta, and gamma oscillations in the human MEG. Finally, some studies have revealed similarities in the scalp topographies and functional characteristics of alpha and beta activity (Chen et al., 2008; Shackman et al., 2010). The present study extends these findings using EEG spectroscopy of a large sample of healthy individuals.

Besides frequencies, we also examine the amplitudes of spectral peaks. These can provide additional evidence for the independence or interdependence of rhythms and allow the thalamocortical mean-field model to be tested further. This model has already been shown to be able to account for various aspects of evoked response potentials (Rennie and Robinson, 2002), onset and dynamics of epileptic seizures (Robinson et al., 2002), and correlation and coherence of EEG and electrocorticographic signals (Robinson, 2003). An extension of this model incorporating the basal ganglia successfully mimicked a number of electrophysiological changes in Parkinson’s disease (Van Albada and Robinson, 2009; Van Albada et al., 2009). Correspondence of amplitude relationships with model predictions would constitute additional evidence for its plausibility.

We perform the analyses partly in the light of aforementioned model of thalamocortical activity, but in a way that would allow the model to be invalidated by the data. The model is fitted to eyes-closed spectra of a large group of healthy subjects, and the model parameters are used to estimate a background spectrum without peaks or troughs. This method balances the dual goals of determining a physiologically realistic background, and not making any prior assumptions about relationships between spectral peaks. Frequencies and amplitudes are then estimated of the empirically measured peaks relative to this background, and their interrelationships are explored.

## 2 Materials and Methods

In this section we describe our data collection, peak fitting, and statistical methods. Section 2.1 describes the subject group, EEG recording procedures, and calculation of spectra. Section 2.2 gives a brief account of the model of thalamocortical activity and its predictions concerning relationships between spectral peaks. Sections 2.3 and 2.4 respectively detail the methods for peak fitting and classification.

### 2.1 Subjects and recordings

The data were eyes-closed resting EEG spectra of 1424 healthy subjects (702 females and 722 males), a subset (95%) of those in Van Albada et al. (2010) and Chiang et al. (2011), where any subjects rejected in that study based on excessive voltage fluctuations at 14 or more electrodes were also excluded here, resulting in the removal of 39 subjects of the original 1463. Subjects’ ages ranged from 6.08 to 86.55 years (mean 26.88 years). The recordings were obtained with a NuAmps amplifier (Neuroscan) by Brain Resource, Ltd. (www.brainresource.com) and made available through the Brain Resource International Database (BRID; Gordon et al., 2005). The montage included 26 electrodes placed according to an extended International 10–20 system (Klem et al., 1999). Of these, we focus on the Cz electrode, which is relatively unaffected by muscle artifact and combines frontal and occipital influences. The sampling rate was 500 Hz and average of mastoids was used as a reference. An analog low-pass filter removed 40 dB per decade above 100 Hz. Data were corrected offline for eye movements using a method based on that of Gratton et al. (1983). The spectrum was calculated from 2 min of relatively artifact-free EEG with a resolution of 0.25 Hz by averaging the spectra of 50% overlapping 4 s epochs after multiplying each epoch’s time series by a Welch window. We compared our findings with 981 spectra that were identical except for the use of a Hann instead of a Welch window, to exclude the possibility of results depending on the particular choice of windowing function.

### 2.2 Thalamocortical model

Background spectra and predictions of peak frequencies and amplitudes were calculated using a mean-field model of thalamocortical electrical activity (Robinson et al., 2001b, 2002, 2003a, 2005). It is beyond the scope of this paper to give a detailed mathematical account of the model, but we introduce some aspects here to clarify theoretical predictions of peak frequencies and amplitudes. Section 2.2.1 gives a brief overview of the model, Section 2.2.2 provides approximate frequencies of corticothalamic resonances, and Section 2.2.3 discusses qualitative predictions on relationships between peak amplitudes. For a more detailed treatment we refer the reader to the papers cited.

#### 2.2.1 Overview of the model

**Figure 2:**
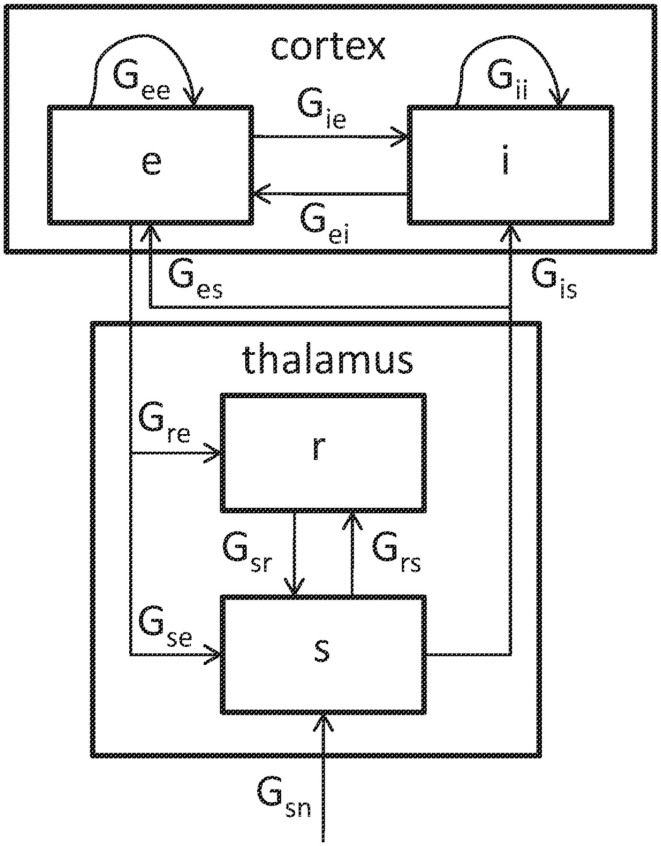
**Schematic representation of the model, including the following populations: *e*, cortical excitatory neurons; *i*, cortical inhibitory neurons; *r*, thalamic reticular nucleus; *s*, primary and secondary thalamic relay nuclei**. In the linearized version of the model, each connection has a gain *G_ab_* (*a*, *b* = *e*, *i*, *r*, *s*). The relay nuclei receive input from the brainstem, indicated by the subscript *n*.

The structure of the model is illustrated in Figure 2. We here consider only the version obtained by linearizing about its fixed-point firing rates. The neural populations included are cortical excitatory (*e*), cortical inhibitory (*i*), thalamic reticular (*r*), and thalamic relay (*s*) including both primary relay and association nuclei. Each population is described by its instantaneous mean firing rate. The *e* and *i* populations connect both to themselves with gains *G_ee_* and *G_ii_*, and to each other with gains *G_ie_* and *G_ei_*, quantifying the change in output rate divided by the change in input rate. Similarly, the relay nuclei project to the cortical populations with gains *G_es_* and *G_is_*. In each case, the second subscript corresponds to the sending population and the first subscript to the receiving population. Approximating connections in the cortex as random leads to *G_ii_* = *G_ei_*, *G_ie_* = *G_ee_*, and *G_is_* = *G_es_* (Braitenberg and Schüz, 1998; Robinson et al., 2001b). Besides cortical interactions, the following loops involving the thalamus are seen: a direct corticothalamic loop passing only through the relay nuclei; an indirect corticothalamic loop also passing through the reticular nucleus; and an intrathalamic loop that involves reciprocal connections between the relay and reticular nuclei. These loops are associated with gains *G_ese_* = *G_es_G*_se_, *G_esre_* = *G_es_G_sr_G_re_*, and *G_srs_* = *G_sr_G_rs_*, respectively.

Spectra can be computed from the model by approximating brainstem input as white noise, and assuming that EEG signals are proportional to the activities of the cortical excitatory neurons (Robinson et al., 1997, 2001a, 2005). Such model spectra were fitted to empirical ones using a fitting procedure that uses a Monte Carlo method with repeated random initializations to avoid finding false minimums (Robinson and Rennie, 2010; Rowe et al., 2004). The quantity minimized was a weighted sum of squared differences between log empirical and log predicted spectra at each frequency. The free parameters were a synaptodendritic time constant *α*, a cortical damping rate *γ*, the corticothalamic axonal latency *t*_0_, an overall scale factor *p*_0_, and the gains *G_ee_*, *G_ei_*, *G_ese_*, *G_esre_*, and *G_srs_*. For further details we refer to the papers cited.

Model spectra consist of a background modulated by thalamocortical interactions yielding peaks and troughs. The background is calculated by retaining projections from thalamus to cortex, but setting the strengths of projections from cortex to thalamus to zero.

#### 2.2.2 Frequency estimates via approximation of the dispersion relation

The thalamocortical model uses a damped-wave equation to describe the propagation of neural activity across the cortical sheet (Robinson et al., 2001b). By Fourier transforming the spatiotemporal model equations, an expression for the activity of the cortical excitatory neurons can be obtained in terms of frequencies and wavenumbers. Equating the denominator of this expression to zero yields a dispersion relation, determining the characteristics of the damped waves making up the activity.

**Figure 3:**
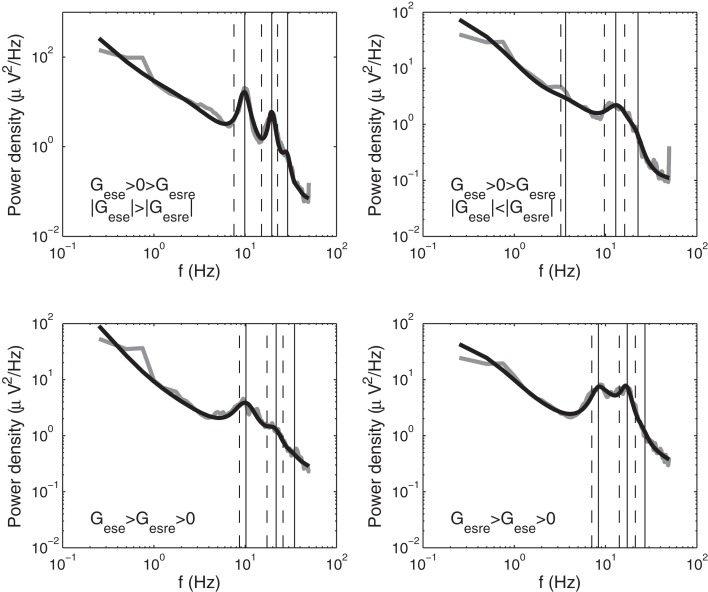
**Initial approximations (dashed vertical lines) and precise estimates (solid vertical lines) of peak frequencies in model spectra for four subjects with different values of their corticothalamic loop gains *G_ese_* and *G_esre_***. For subjects with *G_ese_* > 0 > *G_esre_* and |*G_ese_*| < |*G_esre_*|, we determined peaks in the theta and what we term “iota” and “xi” ranges. For all other subjects, we determined alpha and what we term “beta_1_” and “beta_2_” peaks. For our definitions of iota, xi, beta_1_, and beta_2_, see Figure 7 and Table 1. Empirical spectra are shown in gray, model spectra in black.

In this study we estimated peak frequencies for model spectra in two ways: the first is based on approximations of the dispersion relation for the linearized model, and the second refines these estimates by looking for peaks close to these approximations in background-subtracted model spectra. In the present section, we focus on the approximate frequencies, while the peaks in fitted spectra are described in Section 3.1. Results of these two methods are illustrated in Figure 3.

In general, the dispersion relation has complex angular frequencies *ω* = *ω_x_* + *iω_y_* as solutions, where *ω_x_* determines the oscillation frequency of the solution, and *ω_y_* its temporal damping rate. There are no relevant solutions with *ω_y_* = 0, since instabilities set in at boundaries where the dispersion relation has real solutions. Spectral peaks for real frequencies *ω* = *ω_r_* occur when the dispersion relation is closest to having a zero. Since uniform modes turn out to be the least damped (Robinson et al., 1997, 1998), we consider only the dispersion relation for zero wavenumber:

(1) $$\begin{array}{llll} & {\left(1-\frac{\mathrm{i\omega}}{\gamma }\right)}^{2}-\frac{1}{1-G_{ei}L\left(\omega \right)} & & \\ & \;\times \left[G_{ee}L\left(\omega \right)+\frac{G_{ese}L^{2}\left(\omega \right)+G_{esre}L^{3}\left(\omega \right)}{1-G_{srs}L^{2}\left(\omega \right)}e^{i\omega t_{\text{0}}}\right]=0, \end{array}$$

where *t*_0_ is the thalamocortical axonal loop delay, γ is a damping rate for cortical activity propagation, and *L*(ω) accounts for low-pass filtering of signals in synapses and the dendritic tree,

(2) $$L\left(\omega \right)={\left(1-\frac{\mathrm{i\omega}}{\alpha }\right)}^{-1}{\left(1-\frac{\mathrm{i\omega}}{\beta }\right)}^{-1}.$$

Here, β and α are synaptodendritic rise and decay rates, respectively.

To simplify equation (1) we use the approximations (Roberts and Robinson, 2008)

(3) $$L(\omega )\approx \exp\left[i\omega \left(\frac{1}{\alpha }+\frac{1}{\beta }\right)\right],$$

(4) $${\left(1-\frac{i\omega }{\gamma }\right)}^{2}\approx e^{-2i\omega /\gamma },$$

valid for *ω* < *α*, *β*, *γ*. Dividing equation (1) by the first term, we obtain

(5) $$\begin{array}{l} 1-\frac{1}{\left(1-G_{ei})(1-G_{srs}\right)}(G_{ese}e^{i\omega \left(t_{0}+\frac{2}{\alpha }+\frac{2}{\beta }+\frac{2}{\gamma }\right)}\\ +G_{esre}e^{i\omega \left(t_{0}+\frac{3}{\alpha }+\frac{3}{\beta }+\frac{3}{\gamma }\right)})=0, \end{array}$$

where factors of *L*(*ω*)were retained only in the numerators, and the term involving *G_ee_* was dropped, since it was previously found by numerical exploration to be of secondary importance for peak locations (Robinson et al., 2001b). Peaks occur where equation (5) is closest to being solved, frequencies depending on the relative strengths and signs of direct and indirect thalamocortical feedback. Generally, *G_ese_* > 0, *G_esre_* < 0, and |*G_ese_*| > |*G_esre_*| reflecting the waking state with positive overall thalamocortical interactions and a relatively inactive thalamic reticular nucleus (Robinson et al., 2002, 2004; Van Albada et al., 2010). Minimums of the left-hand side of equation (5) then occur approximately where the complex argument of the *G_ese_* term is 2*πn* (*n* = 1, 2, …). The strongest resonance or putative alpha rhythm corresponds to *n* = 1, leading to the frequency estimate

(6) $$f_{\alpha }\approx \frac{1}{t_{\text{0}}+2∕\alpha +2∕\beta +2∕\gamma }.$$

Peaks in the low and high beta ranges, which we will term beta_1_ and beta_2_ peaks, correspond to *n* = 2 and *n* = 3, and are located around 2 and 3 times the alpha peak frequency. Due to the approximations made, equation (6) tends to underestimate peak frequencies; more precise estimates are made in Section 3.1.

In some cases, *G_ese_* > 0, *G_esre_* < 0, and |*G_esre_*| > |*G_ese_*|, so that thalamocortical resonances arise in an overall negative feedback loop. Peaks then occur where the argument of the *G_esre_* term is *π* + 2*πn* (*n* = 0, 1, …). The first of these resonances is a putative theta rhythm with frequency (Robinson et al., 2002)

(7) $$f_{\theta }\approx \frac{1}{2t_{0}+6∕\alpha +6∕\beta +4∕\gamma }.$$

Note that this is close to half the alpha peak frequency in equation (6) if *t*_0_ + 2/α + 2/β + 2/γ ≫ 1/α + 1/β. In our sample, this was generally the case, the difference between the estimated theta frequency and half the estimated alpha frequency being of order 10%. Higher-order peaks are expected for *n* = 1, 2 with frequencies around 3 and 5 times *f_θ_*, respectively.

Since no hard limit was imposed during fitting to force *G_esre_* to be negative, there were also some cases with *G_ese_* > 0, *G_esre_* > 0. For *G_ese_* > *G_esre_*, the frequency becomes

(8) $$f_{\alpha }\approx \frac{1}{t_{0}+3∕\alpha +3∕\beta +2∕\gamma },$$

in analogy with the previous derivations. Higher-order peaks are expected around integer multiples of this frequency.

We used the estimates equations (6–8) to label peaks in fitted model spectra as theta, alpha, etc., and to obtain more precise predictions of relationships between peak frequencies. For spectra with a theta peak, higher-order peaks are expected to lie between alpha and beta_1_, and between beta_1_ and beta_2_. Following the tradition of denoting EEG rhythms by Greek letters, we refer to these rhythms as iota and xi. The definitions of these bands are illustrated in Figure 7, where different sets of band limits were used depending on the location of the highest peak, as described in Section 2.4.1.

#### 2.2.3 Qualitative predictions of amplitude relationships

The thalamocortical model also predicts the amplitudes of the various peaks to covary. We here provide qualitative predictions of such relationships, while quantitative estimates are obtained from fitted model spectra in Section 3.1.

Since beta peaks arise as near-harmonics of alpha peaks in the model, the prediction of a positive association between their amplitudes is straightforward. Predicting the relationship between theta and alpha peaks is more complicated. Simultaneous theta and alpha peaks in empirical spectra could be due to activity in parallel thalamocortical pathways with different gains, or to temporal changes in gain in a single pathway. For instance, positive net feedback may exist in some regions, with negative feedback in others, especially in the drowsy state near the sleep-wake transition, thereby allowing theta and alpha peaks to coexist. Concurrent peaks in what are traditionally considered the theta and alpha ranges could also arise due to parallel thalamocortical loops with different delays, or due to spatial variations in loop delays (Robinson et al., 2001a, 2003b). The version of the model considered here does not account for concurrent theta and alpha peaks via these mechanisms, due to static gains and the lumping of possible parallel or spatially varying thalamocortical loops into a single loop. However, empirical theta peaks can be considered to be superposed on the model background and on troughs which the thalamocortical model also predicts in this range, as described in the next section.

Correlations between theta and alpha peak amplitudes are expected to have contributions from opposing mechanisms. In our model, positive and negative *G_ese_* + *G_esre_* generally lead to alpha and theta peaks, respectively, and their amplitudes tend to be large when |*G_ese_* + *G_esre_*| is large. The common dependence of *G_ese_* and *G_esre_* on the thalamocortical gain *G_es_* will contribute positively to the correlation between empirical theta and alpha peak amplitudes. If concurrent peaks in what are traditionally labeled the theta and alpha ranges arise due to spatial variations in thalamocortical loop delays, a positive association between their amplitudes is also expected. Note that such peaks should actually be labeled by their generating mechanisms rather than by frequency ranges; however, this is difficult to do in practice based directly on empirical spectra.

In the following, we require that the frequency of theta peaks differ by more than 3 Hz from that of the alpha peak. Since it is possible in principle to have split alpha peaks with a larger frequency difference, alpha splitting may provide a small positive contribution to the relationship between empirical peaks in the nominal theta and alpha ranges determined in this paper.

On the other hand, substantial spatial or temporal variations in *G_ese_* + *G_esre_* are required to produce large alpha peaks at one time or location, and large theta peaks at another. Assuming that small variations are more likely, especially within the limited time window from which spectra are computed, this will provide a negative contribution to the association between alpha and theta peak amplitudes.

### 2.3 Fitting of gaussian peaks

**Figure 4:**
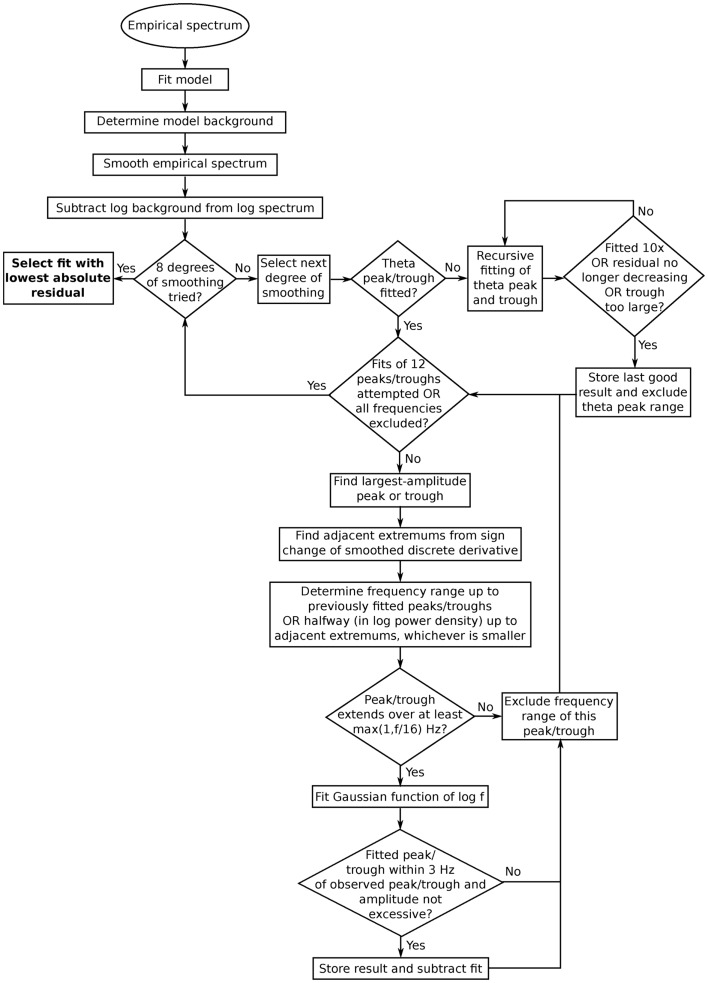
**Flowchart showing the steps involved in fitting peaks and troughs to empirical spectra**. The different degrees of smoothing refer to the step where extremums adjacent to the current peak or trough are found. A low degree of smoothing tends to yield narrow peaks/troughs, whereas a high degree of smoothing tends to yield broad ones. Different degrees of smoothing may be appropriate for different levels of noise. Such smoothing improved the agreement between fitted and visually identified peaks.

The fitting routine is illustrated in the flowchart in Figure 4. It differed in several respects from one previously used to identify alpha peaks in the subjects considered here plus 32 additional subjects (Chiang et al., 2008, 2011). First, the current fitting routine covers not just the alpha frequency range but the larger range 2–35 Hz. Another notable difference is that Chiang et al. (2008, 2011) considered spectra at multiple electrode sites to find clusters of alpha peaks with similar frequencies. Furthermore, in those papers, peaks were fitted with Gaussian functions of log power vs. *f*, whereas we use Gaussian functions of log power vs. log *f*. However, we compared our results with fits of log power vs. *f*, finding no strong differences. The methods also differ in the type of background used: the previous papers considered peaks superposed on a power-law background, while the current paper examines peaks and troughs that modulate a model-based background. This is done to assess spectral features due to thalamocortical interactions (cf., Section 2.2). The model-fitting routine has been validated and its properties analyzed in a number of publications (Rowe et al., 2004; Van Albada et al., 2007, 2010). We do not analyze troughs further in this paper, but including them in the fitting routine enables their future analysis and is relevant for the theta range, as further explained in the following. Finally, the papers cited used a single degree of spectral smoothing, whereas we compared moving averages with different ranges and selected the closest fit, which helped ensure that no peaks were missed, and yielded close agreement with visually identified peaks. The fitting method was developed independently of the results on relationships between peaks described here.

**Figure 5:**
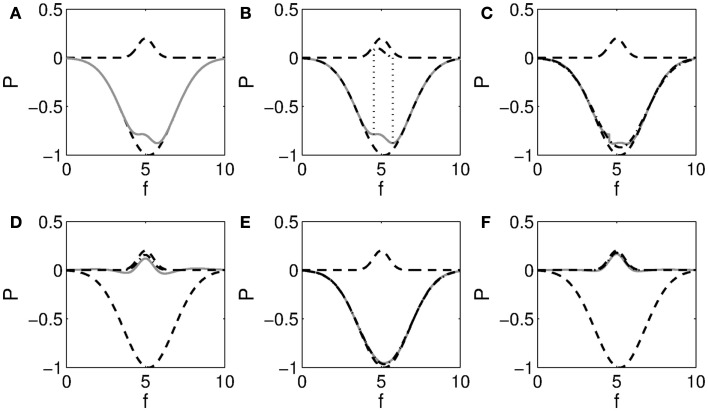
**Illustration of recursive fitting of overlapping peak and trough**. **(A)** Shows a Gaussian peak at *f* = 5 and trough at *f* = 5.2 (dashed), and their sum in gray. A small peak remains visible over the range shown in **(B)**. Fitting this peak leaves the residual shown in gray in **(C)**. The dash-dotted line indicates the trough fitted to this residual. Subtracting this trough yields the residual shown in gray in **(D)**. After two more steps, shown in **(E)** and **(F)**, it is seen that the fitted peak and trough closely match the actual ones.

**Figure 6:**
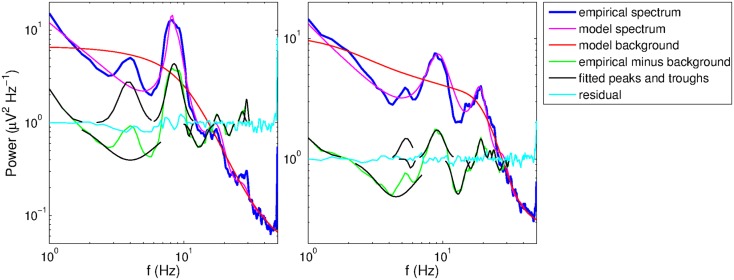
**Example spectra from two subjects with overlapping theta peak and trough**. The residuals and fitted peaks and troughs (in log power) are exponentiated for visual clarity on the logarithmic scale.

Before fitting, spectra were smoothed using a five-point moving average to reduce noise. Up to 12 peaks and troughs were then fitted to the difference of log spectra and log background in the range 2–35 Hz. This number of peaks was chosen since it was seen to adequately capture all visually identified peaks in the range considered. The theta range was fitted first, since background-subtracted empirical spectra suggested that overlapping peaks and troughs were present in this range, and therefore an adjusted method was used for theta. Peaks were first sought in that part of the range 2–9 Hz where the spectrum was below the background, corresponding naively to the theta range. Additional smoothing was then applied until at most a single peak was present in the range. If a peak was present and the distance between its adjacent minimums was ≥1 Hz (to avoid spurious sharp peaks), recursive fitting was performed of the overlapping peak and trough, as illustrated in Figure 5. This entailed the following steps: first, the peak was fitted on a possible constant baseline, and the fitted values were subtracted. Then the trough was fitted with zero baseline, the residual was calculated with only the trough subtracted, and the peak was again fitted. The latter sequence was repeated up to 10 times as long as this decreased the residual computed by subtracting both peak and trough. A further constraint was that the fitted trough was not more negative on average than the empirical one in the first and last quarters of its frequency range. This ensured that recursive fitting did not lead to very large peaks and troughs where these were not present in empirical spectra. Two examples of spectra with overlapping theta peak and trough are given in Figure 6.

The remaining peaks and troughs were fitted in order of decreasing amplitude. Peaks were sought in those ranges where no peaks or troughs had yet been fitted, except for theta, where initially only the range of the peak (the closed range of frequencies between its adjacent minimums) was excluded to allow possible additional peaks in this range to be found. If a frequency range was not bounded by already-fitted peaks or troughs, it was bounded by the closest frequencies where the residual was of opposite sign to that of the extremum. To avoid fitting spurious narrow peaks, only those peaks or troughs were considered that extended over at least max(1, *f*/16) Hz, where *f* is the frequency of the extremum in Hz. Locations of extremums were identified using eight different degrees of smoothing (but the same degree for each peak/trough), and the fit with the lowest absolute residual was selected. The limit to the number of peaks and troughs fitted prevented under-smoothing, resulting in approximately uniformly distributed degrees of smoothing. Gaussian peak or trough values were subtracted over the range where they were ≥0.05 and ≤−0.05, respectively. This fitting algorithm yielded good agreement with visually identified peaks and troughs.

### 2.4 Peak classification

Frequency bands for the analysis of peak parameters are defined in Section 2.4.1. Peak classification took into account putative split alpha and beta peaks, as explained in Section 2.4.2, but the detailed analysis of split peaks is left to future work considering multiple electrodes. Peak exclusion criteria are described in Section 2.4.3. These take into account the statistical nature of EEG spectra, eliminating peaks that may have occurred by chance.

#### 2.4.1 Band limits

**Figure 7:**
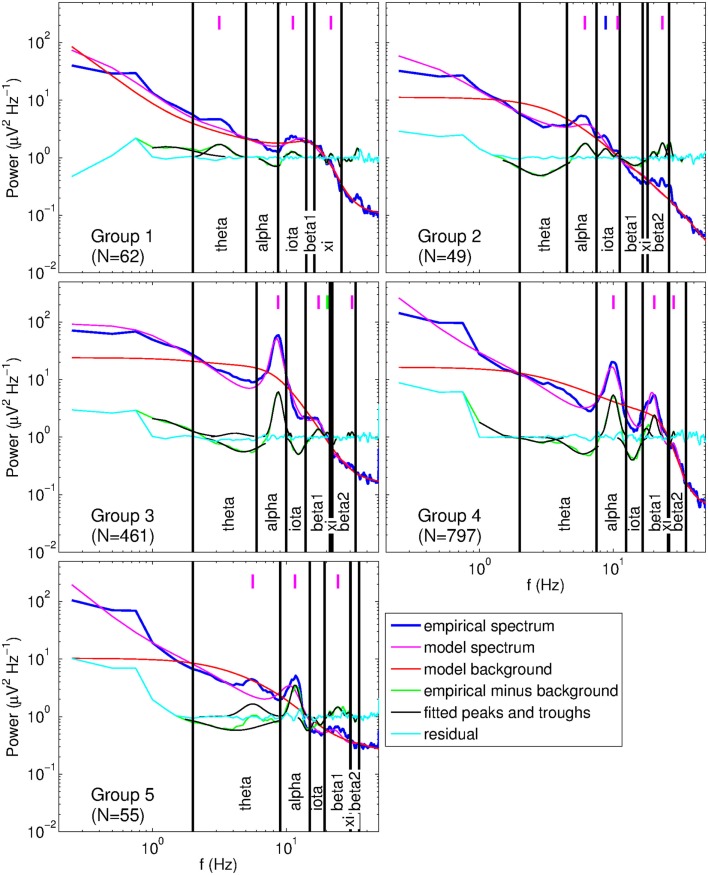
**Example spectra of subjects from each group having a different set of band limits, as listed in Table 1**. Vertical lines indicate peak locations: magenta, primary peaks; blue, secondary alpha peak; green, secondary beta_1_ peak.

**Figure 8:**
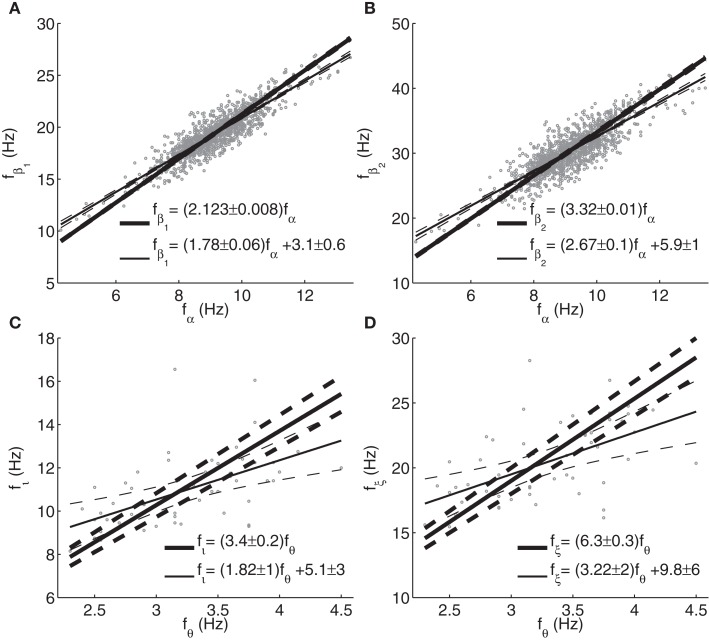
**Relationships between frequencies of peaks or spectral enhancements determined from fitted model spectra**. **(A)** Beta_1_ vs. alpha; **(B)** beta_2_ vs. alpha; **(C)** iota vs. theta; **(D)** xi vs. theta. Thick lines, fits of frequency ratios; thin lines, linear fits with intercept. Dashed lines represent 99% non-simultaneous confidence intervals for the linear trends, and the corresponding 99% confidence bounds for the slopes and intercepts are indicated. Note that beta_2_ frequencies can exceed 35 Hz (the maximum frequency of fitted Gaussian peaks), since model spectra were evaluated up to 50 Hz.

We defined band limits based on the location of the largest peak in the range 2–13 Hz. To prevent influencing correlations by the choice of band limits, we assigned subjects to five groups with appropriate band definitions, and analyzed correlations for each group separately. Figure 7 gives example spectra with fits from each group and illustrates the corresponding bands. If the largest peak was in the range 2–5 Hz (Group 1, *N* = 62) it was considered to be a theta peak, and if it was in the range 5–13 Hz it was treated as an alpha peak. A further subdivision was made based on alpha peak frequency: 5–7 Hz (Group 2, *N* = 49), 7–9 Hz (Group 3, *N* = 461), 9–11 Hz (Group 4, *N* = 797), and 11–13 Hz (Group 5, N = 55). Symmetric bands were defined around these 2-Hz ranges, bandwidth increasing with alpha peak frequency (by 1 Hz for each consecutive group) to maximize coverage of the frequency space. Bands were then defined via the linear regression equations for peak frequencies derived from fitted model spectra (cf., Figure 8). For Group 1, iota and xi limits were calculated from theta limits, while for Groups 2–5, beta_1_ and beta_2_ limits were calculated based on the alpha band. As a result, the iota and xi bands were relatively wide for subjects whose main peak was a theta peak, while the beta_1_ and beta_2_ bands were relatively wide for subjects whose main peak was an alpha peak. This helped ensure that no peaks were missed in the relevant bands. The resulting bands are listed in Table 1. Correlations between peak parameters were determined using only the largest peak in each band.

**Table 1:** **Frequency bands in Hz, based on the frequency of the largest peak in the range 2–13 Hz**.

Group	Theta	Alpha	Iota	Beta_1_	Xi	Beta_2_
1	2–5	5–8.7	8.7–14.2	14.2–16.3	16.3–25.9	–
2	2–4.5	4.5–7.5	7.5–11.1	11.1–16.4	16.4–17.9	17.9–25.9
3	2–6	6–10	10–13.8	13.8–20.9	20.9–21.9	21.9–32.5
4	2–7.5	7.5–12.5	12.5–16.4	16.4–25.3	25.3–25.9	25.9–35
5	2–9	9–15	15–19.1	19.1–29.8	29.8–29.9	29.9–35

*The following ranges were distinguished for the largest peak: 2–5 Hz (Group 1), 5–7 Hz (Group 2), 7–9 Hz (Group 3), 9–11 Hz (Group 4), and 11–13 Hz (Group 5). Note that the Gaussian peaks are fitted curves and hence not constrained by the 0.25 Hz resolution of the empirical spectra*.

#### 2.4.2 Split peaks

Peaks in the range 5–13 Hz, differing from the primary alpha peak by no more than 3 Hz and less than a factor of two in height, were considered to be secondary alpha peaks. If there were several peaks fulfilling these criteria, the one with the smallest frequency difference with the primary peak was chosen. If such a peak was the highest in the theta or iota band, the next-highest peak in the relevant band was taken to be the primary theta or iota peak, if present.

Secondary beta_1_ peaks were considered to be those peaks lying within 6 Hz of the primary beta_1_ peak, at higher frequency than the highest-frequency alpha peak and not directly flanking it, and differing by less than a factor of two in height from the primary peak. As for alpha, if several such peaks were present, the one closest to the primary peak was selected. If the secondary beta_1_ peak fell outside the beta_1_ band, the next-highest peak in the relevant band was considered to be the primary peak for that band.

This classification of split peaks may be refined and further analyzed in future studies using data from multiple electrodes, as done for alpha peaks by Chiang et al. (2008, 2011).

#### 2.4.3 Rejection criteria

Peaks in the theta or iota bands were rejected if they immediately flanked alpha peaks (using the criterion that their frequency ranges had an overlap of at least two points, corresponding to a range of 0.25 Hz) and were more than four times smaller than the alpha peak, since such peaks usually appeared to result from non-Gaussianity of the alpha peak. The rejected peak was replaced by the next-highest peak in the same range, if present.

Another criterion for peak identification was a good signal-to-noise ratio. At each frequency, a nine-point root mean square deviation between log raw and log smoothed spectra was determined as an estimate of noise. The 10% of peaks with the lowest ratio of height to this RMS deviation at the nearest frequency point were rejected. It is of course possible that some spurious peaks were nevertheless fitted, but these will be randomly scattered and not influence the main trends.

In some cases model spectra did not closely fit empirical spectra. After classifying peaks into bands, we therefore determined the mean deviation between log empirical and log model spectra, and excluded peaks when the model fit was among the worst 15% for the given group and band. Visual inspection showed this to be a relatively conservative exclusion criterion, so that only peaks were considered where the model fit well. Mean deviations rather than mean absolute deviations were used because the reliability of the background depends mainly on whether the model fit is systematically above or below the empirical spectrum. This criterion was not applied to the theta band, since model fits did not yet adequately capture theta peaks. Instead, theta peaks were rejected if the fit in the alpha band was among the worst 15%. Note that empirical theta peaks could nevertheless be investigated, since the model background was fitted in this range, and Gaussian theta peaks and troughs were fitted on top of this background, as explained in Section 2.3.

The rejection criteria were chosen to obtain a maximal set of fitted peaks showing good correspondence with visually identified peaks. Thus, the criteria were independent of the results reported here. The qualitative results were robust to variations of rejection levels.

## 3 Results

Section 3.1 concerns relationships between peak frequencies and heights found from fitted model spectra. These may be regarded as theoretical predictions using physiologically realistic parameters, and as such are intermediate between theoretical predictions and empirical results. The limited number of model parameters prevents overfitting and ensures that relationships between model peaks do not simply reflect empirical ones. The results for Gaussian peaks fitted to empirical spectra are discussed in Section 3.2.

### 3.1 Peak relationships based on fitted model spectra

The following two sections respectively describe the frequency and amplitude relationships of peaks in fitted model spectra.

#### 3.1.1 Frequency relationships

Figure 8 shows the dependences of beta_1_ and beta_2_ frequencies on alpha frequency, and of iota and xi on theta frequency, where peaks were labeled as described in Section 2.2. Spectra (including background) with *G_ese_* + *G_esre_* < 0 often showed a theta enhancement but no actual theta peak. Therefore, theta frequencies were determined from sign changes of the second derivative of the spectrum with respect to frequency. We excluded those cases from analysis where the spectrum was below the background at the theta frequency thus determined (9 out of 64). It is seen that theta peaks or shoulders in model spectra tend to occur much below half the normal alpha peak frequency. This may be an artifact due to the fact that the fitted version of the model has only a single set of gains and therefore does not account for concurrent theta and alpha peaks. Thus, cases with *G_ese_* + *G_esre_* < 0 have theta and iota peaks in fitted spectra, with a frequency ratio close to three. Since the fitting routine emphasizes the goodness of fit for the peak around 10 Hz, theta peaks are fitted around 3 Hz even when they empirically occur around 5 Hz. In contrast, the analysis in Section 2.2 showed that variations in thalamocortical gains will tend to cause theta and alpha peaks with a frequency ratio close to two. We will take this as our prediction of the relationship between theta and alpha peak frequencies.

The mean ratios of beta_1_ and beta_2_ peak frequencies to the alpha peak frequency were 2.123 ± 0.008 and 3.32 ± 0.01, respectively, slightly above the ratios of 2 and 3 predicted based on the approximations in Section 2.2. Due to the non-zero intercepts of the linear trends, these ratios depend somewhat on the constitution of the sample. For instance, for the 50% of subjects with the lowest alpha peak frequencies, the mean ratios were closer to 2.2 and 3.4. The iota-to-theta frequency ratio was 3.4 ± 0.2, again somewhat above the approximate theoretical prediction of 3. Similarly, the xi-to-theta ratio was 6.3 ± 0.3, to be compared with the value of 5 based on the simplified equations in Section 2.2. We note that the reliability of the latter estimates is somewhat compromised by the possible fitting of iota peaks to actual alpha peaks, as mentioned above, which may have affected the parameter values and hence relative peak locations.

#### 3.1.2 Amplitude relationships

**Figure 9:**
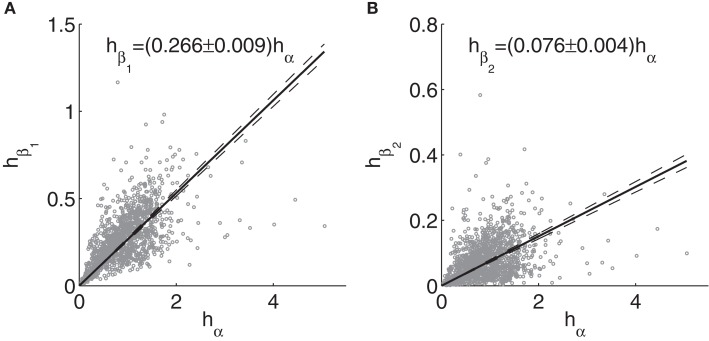
**Relationships between peaks heights from fitted model spectra**. **(A)** Beta_1_ vs. alpha; **(B)** Beta_2_ vs. alpha. Dashed lines and the text indicate 99% confidence intervals for the fits.

Relationships between alpha and beta peak heights are illustrated in Figure 9. The regressions were performed without an intercept term, since no beta peaks arise in the model without alpha peaks. The heights are more scattered than the frequencies, but clear positive trends remain. The slopes of the trend lines are slightly reduced by the few spectra with particularly strong alpha, even though the two spectra with the highest alpha peaks in fitted spectra were excluded, since fitted peaks did not accurately reflect empirical ones in these cases. For instance, excluding all cases with alpha height >3, the slopes become 0.278 and 0.079 for beta_1_ and beta_2_, respectively.

No significant correlations were found between theta peak heights on the one hand and iota and xi peak heights on the other hand (*p* > 0.5). However, iota and xi peak heights did have a positive association (*r* = 0.63, *p* = 9.9*e* − 8), with the slope of the regression line for xi vs. iota height being 0.4 ± 0.1. A similar word of caution applies to these amplitude relationships as to the corresponding frequency relationships, since the model parameters for subjects with *G_ese_* + *G_esre_* < 0 may be affected by the simultaneous presence of theta and alpha peaks in empirical spectra.

### 3.2 Empirical peak relationships

Here we respectively present the empirical findings on frequency and amplitude relationships between spectral peaks, and compare these with the model predictions.

#### 3.2.1 Frequency relationships

**Figure 10:**
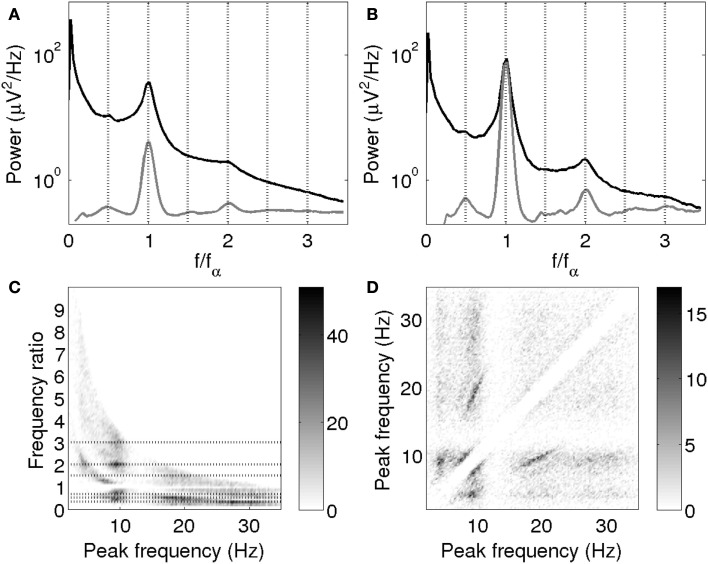
**(A,B)** Mean empirical spectrum (black) and fitted peaks (gray) vs. *f*/*f_α_*. **(A)** All subjects for which alpha peak parameters were obtained. **(B)** The 10% of subjects with the highest alpha peak amplitudes. Fitted peaks are scaled for clarity, with the same scale in **(A,B)**. **(C)** Ratios of all pairs of peak frequencies within spectra. Dotted lines are drawn at 1/3, 1/2, 2/3, 3/2, 2, and 3. **(D)** Pairs of peak frequencies within spectra. The grayscales indicate the density of points. The empty diagonal band reflects the omission of the 1:1 points and the separation necessary for peak resolution.

Figure 10 shows the average empirical spectrum and average fitted Gaussian peaks of log power vs. log frequency plotted against *f*/*f_α_*, where *f_α_* is the individual alpha frequency; this permits frequency ratios to be explored. Averages consisted of mean spline-interpolated spectra across those subjects for which an alpha peak was fitted and not rejected based on the model fit.

It is clearly seen that beta_1_ peaks occurred on average close to twice the alpha peak frequency, while theta peaks occurred around half the alpha peak frequency. Third harmonics of alpha may have been too small or scattered to be visible in the overall average. Since we expect large alpha peaks to be concurrent with large beta peaks, we also plotted the average for the 10% of subjects with the largest alpha peaks separately. This average does seem to have a shoulder around three times the alpha peak frequency (Figure 10B). The average fits additionally show small peaks around 1.5 times the alpha peak frequency, which are however not clearly apparent in the mean empirical spectra. This effect might be explained by the presence of superposed positive and negative power modulations.

Figures 10C,D provide a visualization of these findings that avoids labeling peaks as “alpha” or otherwise, and does not depend on band limits. Figure 10C shows the frequencies of all peaks not rejected based on signal-to-noise ratio vs. their ratios to all other peaks in the same spectrum. Figures 10C,D confirm the association of peaks around 10 Hz with peaks at half, twice and three times that frequency. In particular, the horizontal stripes around (20, 1/2) and (30, 1/3) in Figure 10C clearly show the presence of second and third harmonics of alpha. The constant ratios indicate that these frequencies covary on an individual basis. The individual covariation of theta and alpha frequencies is somewhat less clear, but on average, theta peaks occurred close to half the alpha frequency. Pairs of peaks around 8 and 10 Hz are also seen, possibly representing split alpha. The finite width of the diagonal band arises because a certain minimum separation was necessary in order to resolve peaks; this does not imply a discontinuity in the frequencies of rhythms that can co-occur. The slopes of the frequency relationships are brought out in Figure 10D. The Hann-windowed spectra and the log-linear fits of the Welch-windowed spectra showed the same progression of peak frequencies as the log-log fits of the Welch-windowed spectra.

**Figure 11:**
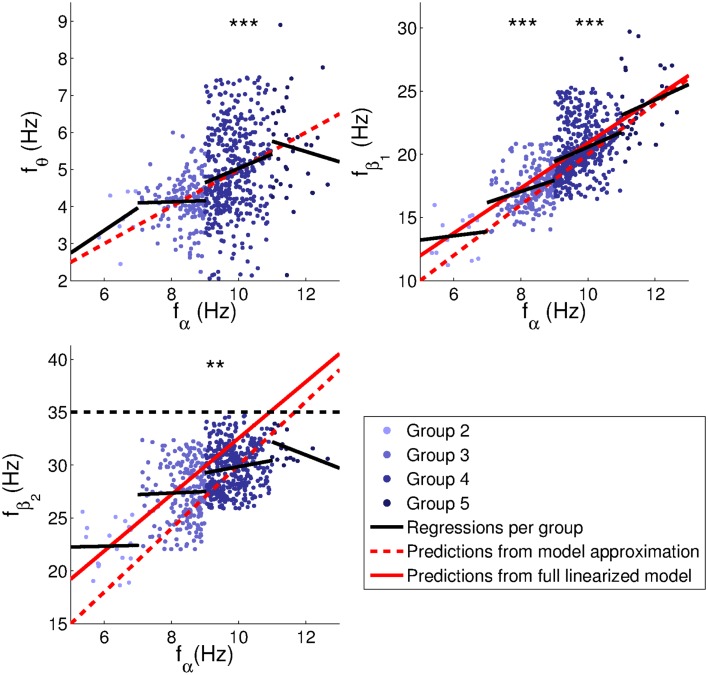
**Relationships between peak frequencies**. Linear regressions were performed for each group separately to avoid spurious correlations induced by the adjustment of band limits to alpha peak frequencies. Correlation coefficients and parameters of the fits are indicated in Table 2. Dashed red lines indicate model predictions based on the approximations in Section 2.2 (*f_θ_* = *f_α_*/2, $f_{\beta_{1}}=2f_{\alpha },f_{\beta_{2}}=3f_{\alpha }$); continuous red lines linear fits with intercepts based on fitted model spectra from Section 3.1. The dashed black line is a reminder that no peaks were fitted above 35 Hz. Significance levels: **0.01, ***0.001.

The relationships between peak frequencies are further illustrated in Figure 11, and Table 2 lists the corresponding correlation coefficients and fit parameters. The plots show only those subjects whose main peak was an alpha peak (Groups 2–5), in order to have only a single set of band limits for each range of alpha frequencies. Intercepts are included since these were found to be significant for both empirical and model peaks in many cases, and since fits without intercept would mainly reflect band limits.

**Table 2:** **Correlation coefficients, the corresponding *p*-values, slopes, and intercepts for linear fits of theta, beta_1_, and beta_2_ peak parameters vs. alpha peak parameters**.

Group	Band	Pearson *r*	*p*-value	Slope	Intercept
**FREQUENCY**					
1	Theta	0.32	0.070	0.3 ± 0.4	2 ± 3
	Beta_1_	0.61	0.061	0.3 ± 0.5	13 ± 4
	Beta_2_	–	–	–	–
2	Theta	0.33	0.39	1 ± 2	0 ± 20
	Beta_1_	0.11	0.70	0 ± 3	10 ± 20
	Beta_2_	0.018	0.94	0 ± 3	20 ± 20
3	Theta	0.022	0.78	0.0 ± 0.3	4 ± 2
	Beta_1_	0.28	2.3*e* − 5***	0.9 ± 0.5	10 ± 4
	Beta_2_	0.025	0.72	0 ± 1	26 ± 9
4	Theta	0.17	0.00037***	0.4 ± 0.30	1 ± 3
	Beta_1_	0.29	3.0*e* − 11***	1.2 ± 0.4	9 ± 4
	Beta_2_	0.14	0.0055**	0.6 ± 0.5	24 ± 5
5	Theta	−0.095	0.58	0 ± 1	10 ± 20
	Beta_1_	0.23	0.18	1 ± 2	10 ± 30
	Beta_2_	−0.41	0.092	−1 ± 2	50 ± 20
**AMPLITUDE**					
1	Theta	0.34	0.050	1 ± 2	0.2 ± 0.5
	Beta_1_	0.0073	0.98	0 ± 4	0 ± 1
	Beta_2_	–	–	–	–
2	Theta	−0.44	0.21	−0.1 ± 0.3	0.3 ± 0.2
	Beta_1_	−0.083	0.78	0.0 ± 0.3	0.4 ± 0.2
	Beta_2_	−0.23	0.34	−0.1 ± 0.2	0.4 ± 0.2
3	Theta	0.29	1.6*e* − 4***	0.12 ± 0.08	0.2 ± 0.1
	Beta_1_	0.35	7.9*e* − 8***	0.12 ± 0.05	0.24 ± 0.07
	Beta_2_	0.14	0.041*	0.04 ± 0.06	0.28 ± 0.07
4	Theta	0.021	0.66	0.01 ± 0.04	0.37 ± 0.06
	Beta_1_	0.36	5.5*e* − 17***	0.12 ± 0.04	0.24 ± 0.06
	Beta_2_	0.18	1.8*e* − 4***	0.05 ± 0.04	0.23 ± 0.05
5	Theta	0.55	4.6*e* − 4***	0.2 ± 0.2	0.2 ± 0.2
	Beta_1_	0.38	0.022*	0.2 ± 0.2	0.2 ± 0.2
	Beta_2_	0.44	0.066	0.1 ± 0.1	0.2 ± 0.1
1–5	Theta	0.039	0.31	0.01 ± 0.04	0.37 ± 0.05
	Beta_1_	0.35	2.7*e* − 24***	0.11 ± 0.03	0.25 ± 0.04
	Beta^2^	0.14	2.4*e* − 4***	0.04 ± 0.03	0.26 ± 0.04

The 99% confidence intervals for the slopes and intercepts are indicated. The numbers of figures reported are adapted to the uncertainties. Significance levels: *0.05, **0.01, ***0.001.

Theta, beta_1_, and beta_2_ frequencies of Group 4 (alpha frequencies in the range 9–11 Hz) had significant positive correlations (at the 0.05 level) with alpha peak frequencies. The theta trend line for this group is close to the theoretical prediction of *f_θ_* = 0.5*f_α_*. Group 3 (alpha frequencies in the range 7–9 Hz) also showed a significant positive correlation between alpha and beta_1_ frequencies. The same correlations were significant for the Hann-windowed spectra, apart from a positive theta-alpha correlation for Group 3 but not Group 4. Using log-linear instead of log-log fits of Welch-windowed spectra also yielded the same pattern of trends, except none of the theta-alpha correlations reached significance. However, all these correlations were positive.

The slopes of the beta_1_ trends for Groups 3 and 4 were 0.9 ± 0.5 and 1.2 ± 0.4, respectively. However, these slopes are affected by the rectangular sampling regions defined by the group-specific band limits, causing many points to lie to the top left and bottom right of a central region of higher density. The slope of this region is very close to 2, matching predictions based on the approximations in Section 2.2. The prediction based on peaks in fitted model spectra (cf., Section 3.1) yields beta_1_ frequencies slightly above the high-density region, and thus seems to be a somewhat poorer fit to the data.

For beta_2_ frequencies, we note that the model-based predictions may be better than they appear visually, since no empirical peaks were fitted above 35 Hz, producing a selection effect. Higher upper limits for the beta_2_ band might therefore have yielded additional points in the upper right-hand corner of the plot, giving a closer correspondence between empirical peak frequencies and model predictions. The Hann-windowed spectra and the log-linear fits of the Welch-windowed spectra showed the same significance levels as the log-log fits of the Welch-windowed spectra for both beta_1_-alpha and beta_2_-alpha frequency correlations.

As noted, the relationships between peaks in fitted model spectra are influenced by the empirical data themselves. The corresponding predictions may be considered as theoretical predictions with physiological parameter distributions, yet the findings should be interpreted with caution. To achieve a level of prediction intermediate between the parameter-independent ones from the approximations in Section 2.2, and the ones from fitted model spectra, we considered model spectra based on independent Gaussian distributions for the model parameters (e.g., gains and delays) with the empirical means and standard deviations, thus destroying any true correlations between the model parameters. This yielded an approximate mean alpha:beta_1_:beta_2_ frequency ratio of 1:2.2:3.8, exceeding both the empirical ratios and the model predictions with correlated parameters. This implies that correlations between the parameters are important for the model to reproduce the empirical frequency relationships.

#### 3.2.2 Amplitude relationships

**Figure 12:**
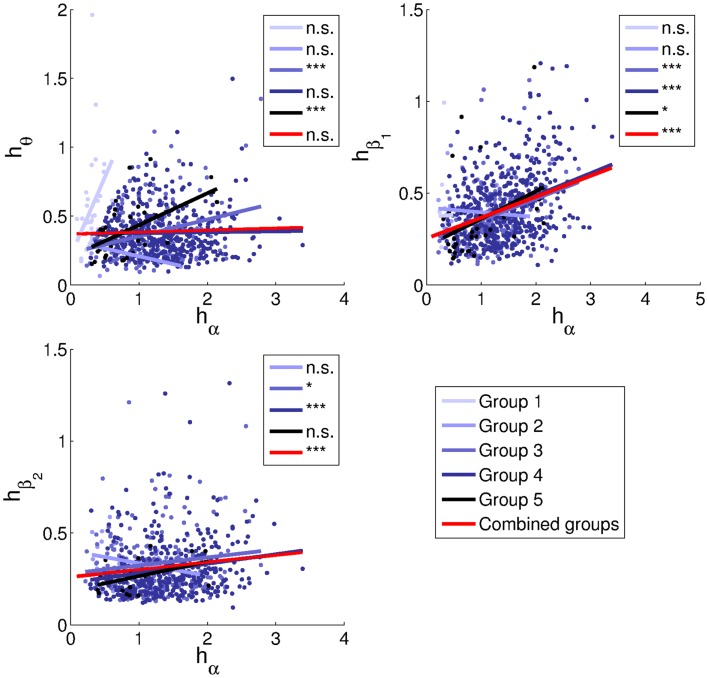
**Relationships between peak heights**. Linear least-squares fits are indicated by lines in the corresponding colors for each group, and in red for all subjects combined. No beta_2_ peaks were identified for Group 1. Correlation coefficients and parameters of the fits are listed in Table 2. Significance levels: n.s. non-significant, *0.05, ***0.001.

Figure 12 shows relationships between peak heights in the different bands, both differentiating between groups and for the sample as a whole. Note that for generality an intercept term was included in the regressions, in contrast to Figure 9. An *F*-test revealed that the intercept significantly improved each of the three whole-sample fits (*p* ≪ 0.001). However, for direct comparison with Figure 9, we also considered fits without intercept.

Alpha and theta peak heights of the combined groups lack a positive relationship. This matches the trend for Group 4 (alpha frequencies in the range 9–11 Hz), while Groups 3 and 5 have significant positive trends.

More convincing positive correlations are seen for beta_1_, being significant for Groups 3–5 as well as for the sample as a whole. The overall slope is 0.11 ± 0.03. Discarding the intercept, the slope is 0.28 ± 0.01, consistent with the prediction of 0.266 ± 0.009 based on model fits.

The overall correlation between beta_2_ and alpha peak heights is 0.14 (*p* = 2.4*e* − 4). For beta_2_ peaks, the slopes are 0.04 ± 0.03 and 0.21 ± 0.01 with and without inclusion of the intercept, respectively, thus bracketing the predicted value of 0.076 ± 0.004. The beta_2_ trends are significantly positive for Groups 3 and 4, and similar in slope to each other and to the trend for Group 5.

The large variability of trends in theta peak height may be partly due to the requirement that theta peaks be higher than alpha peaks for Group 1 and vice versa for Groups 2–5. This constitutes a selection effect that may have increased the slopes of all trend lines, but that would have been strongest for Group 1, due to alpha peaks generally being higher than theta peaks. For Group 5 (alpha frequencies in the range 11–13 Hz), the positive trend may be partly explained by actual alpha and beta_1_ peaks being mislabeled respectively as theta and alpha peaks in a small proportion of cases. Thus, the definition of alpha as corresponding to the largest peak in the range 5–13 Hz may not be optimal, and it could for instance help to take subjects’ ages into account (Van Albada et al., 2010; Chiang et al., 2011). All in all, the relation between alpha and theta peak heights merits further investigation.

Peak height correlations for Hann-windowed spectra differed from those for Welch-windowed spectra for some groups and bands, but for the combined groups, the theta-alpha correlation was still insignificant, while beta_1_-alpha and beta_2_-alpha correlations were positive and highly significant. Moreover, for those cases where the significance levels differed greatly (theta height of Group 1 and beta_2_ height of Group 4), the linear trends were nevertheless quite similar. The same held for the log-linear fits of the Welch-windowed spectra.

We checked whether the positive overall associations between alpha and beta peak heights could be due to relationships between fit deviations in each band. The partial correlation between alpha and beta_1_ peak heights, corrected for deviations between log empirical and model spectra in both bands, is 0.33, close to the uncorrected correlation. However, the corrected correlation between alpha and beta_2_ peak heights is only 0.036. The positive correlation between fit deviations in these bands (*r* = 0.15) may itself be partly due to positively correlated peak heights, but this is impossible to verify without an objectively appropriate background subtraction.

Using independent Gaussian model parameter distributions with the empirical means and standard deviations, model spectra exhibited greater relative beta_1_ and beta_2_ amplitudes, the slopes of the fits without intercept being 0.35 for beta_1_ and 0.15 for beta_2_. This provides a poorer match to the empirical results for beta_1_ but a better match for beta_2_.

## 4 Discussion

Using a large sample (1424) of resting eyes-closed EEG spectra, we have shown clear interdependences between the frequencies and amplitudes of peaks in different bands in this condition, frequencies of many peaks following an approximately harmonic progression. These results strongly suggest that a common process contributes to the different rhythms.

Our main findings are: (i) a positive correlation between theta and alpha peak frequencies for subjects with alpha peak frequencies in the range 9–11 Hz, theta peaks occurring on average near half the alpha peak frequency for the sample as a whole; (ii) peaks in the low beta range tending to occur near twice the alpha peak frequency on an individual-subject basis, with positive correlations between frequencies of alpha and low beta peaks reaching significance for subjects with alpha peak frequencies in the range 7–11 Hz; (iii) peaks in the high beta range tending to occur near three times the alpha peak frequency on an individual-subject basis, with positive correlations between frequencies of alpha and high beta peaks reaching significance for subjects with alpha peak frequencies in the range 9–11 Hz; (iv) a lack of correlation between theta and alpha peak amplitudes for the sample as a whole; and (v) a positive, approximately linear, relationship between alpha and beta_1_ peak amplitudes for the sample as a whole. A positive correlation between alpha and beta_2_ peak amplitudes was also found, but further tests are needed to verify this result.

The harmonic progression of peak frequencies closely matches predictions based on an approximation of a linearized mean-field model of thalamocortical activity (Robinson et al., 2001b, 2005). It is not consistent with any of the following proposals: (i) a geometric progression with a peak spacing of Euler’s number (Penttonen and Buzsáki, 2003; Buzsáki and Draguhn, 2004) or the golden ratio (Roopun et al., 2008a,b; Pletzer et al., 2010); (ii) pacemakers that would not *a priori* be related in frequency or occurrence; (iii) Nunez’s theory of purely cortical eigenmodes, which predicts a non-harmonic sequence of peaks (Nunez, 1995). More generally, to our knowledge there is no model of purely cortical oscillations that predicts the observed peak relationships.

In view of the predicted relationships between peak frequencies, we adjusted band limits to the alpha peak frequency for peak classification. These limits could have been set separately for each subject, followed by a statistical analysis attempting to correct for this. However, to more strongly control for the effects of band limits, we defined only five sets of band limits and investigated trends in each group separately. This yielded group-specific frequency relationships that only reached significance for subjects with alpha peak frequencies in the range 7–11 Hz, probably related to the fact that these were the largest subject groups. It may be investigated in future studies whether larger sample sizes or different band definitions yield significance and similar slopes also in the other groups.

Relationships between peak amplitudes were in good agreement with predictions based on physiological considerations and model spectra. We identified possible contributions to both positive and negative associations between theta and alpha peak amplitudes, consistent with the overall lack of correlation found. Ratios between alpha, beta_1_, and beta_2_ peak heights were close to those from fitted model spectra. Since the latter were partly influenced by the data themselves, we conclude that the empirical results match the model predictions at least semiquantitatively. Amplitude relationships, especially those between alpha and theta peaks, displayed variability between groups of subjects with different alpha peak frequencies. We discussed possible confounding effects that may account for this, suggesting a closer investigation of the theta band in particular.

According to the thalamocortical model, the observed peaks are largely explained by two scenarios: either the inhibitory thalamic reticular nucleus is weakly active, creating a positive thalamocortical feedback loop; or it is strongly active, creating a negative feedback loop. In the first case, the lowest-frequency resonance gives rise to an alpha peak corresponding to one pass through the loop, while in the latter case, it produces a theta peak corresponding to two passes. These basic rhythms are associated with near-harmonics around odd numbers of times the theta frequency, and integer numbers of times the alpha frequency. This agrees with the observed peaks around twice and three times the alpha peak frequency, and the hints of peaks around three times the theta peak frequency.

The covariation of peak frequencies suggests that band limits should be adjusted on an individual basis (at least for the resting-state condition considered here), as also proposed for instance by Doppelmayr et al. (1998) and Klimesch (1999), and consistent with age-associated changes in alpha peak frequency (Van Albada et al., 2010; Chiang et al., 2011) and various theoretical predictions (Nunez and Srinivasan, 1981; Nunez, 1995; Robinson et al., 2001b). It seems most expedient to base the limits on the alpha peak frequency, provided of course that an alpha peak is present. Consistent with the present study, Doppelmayr et al. (1998) argued for a positive association between bandwidth and alpha peak frequency. They measured task-related increases in theta and decreases in alpha peak power, and defined a transition frequency between ranges of increase and decrease. Individual alpha frequency was positively correlated not only with this transition frequency, but also with the difference between the alpha and transition frequencies. Thus, task-related activity confirms that higher alpha peak frequencies imply wider and higher-frequency EEG bands.

**Figure 13:**
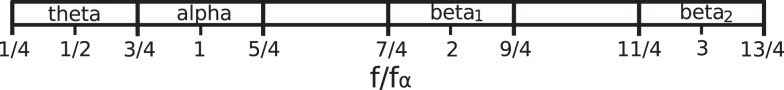
**Proposed band limits based on the frequency of the alpha peak**.

The present theoretical (cf., also Robinson et al., 2001b) and empirical results suggest that, for peak identification, band limits may be placed at approximately *n* + 1/4 and *n* + 3/4 times the individual alpha peak frequency (*n* = 0, 1, …), with theta and low beta peaks respectively being sought in the ranges 1/4–3/4 and 7/4–9/4 times the alpha peak frequency (see Figure 13). This follows especially from results where we avoided classifying peaks and examined pairs of peak frequencies within spectra. This clearly showed that peaks around 20 Hz often occurred together with peaks around half that frequency, and that peaks around 30 Hz often occurred together with peaks around one-third that frequency. These ratios were nearly constant despite individual variations in absolute frequencies. After peak classification, points also clustered around $f_{\beta_{1}}=2f_{\alpha }$ in those subjects with alpha peak frequencies in the range 7–11 Hz. The relationship between theta and alpha frequencies was slightly less clear, but theta peaks occurred on average very close to half the alpha peak frequency. Moreover, for the one group of subjects having a highly significant correlation between alpha and theta peak frequencies (those with alpha peak frequencies of 9–11 Hz), the trend line was close to *f_θ_* = *f_α_*/2. Further research could ascertain whether the bands depicted in Figure 13 are appropriate for detecting task- or state-related changes.

The strictly individual adjustment of frequency bands is appropriate for within-subject comparisons where the alpha peak frequency is relatively stable. It may also be used for group comparisons when the distribution of band limits does not differ systematically between groups. However, depending on the questions asked, individual band adjustment may complicate analyses, since the band limits (and possibly associated filter characteristics, for instance when using wavelet analysis) affect spectral band power, peak characteristics, and the structure of the corresponding oscillations in the time domain. One option for dealing with this can be to define several subgroups, each with fixed frequency bands, as done in the present study. Alternatively, one could correct or account for differences in band definitions, for instance using analysis of variance where band limits constitute one of multiple factors.

Defining algorithms for peak fitting and classification naturally involves many choices that may influence the results. However, fitting Gaussian functions of frequency and of log frequency yielded qualitatively identical results. Moreover, we designed our methods to yield good agreement with visually identified peaks, and we consider it likely that any algorithm fulfilling this criterion will give similar results. This may be further investigated in future studies.

We allow for the possibility of contributions to the EEG which do not conform to the simple pattern of (sub)harmonics of alpha due to thalamocortical resonances. These may include cortically generated rhythms, rhythms originating in the hippocampus or amygdala, and intrathalamically generated rhythms such as sleep spindles (Robinson et al., 2002; Niedermeyer and Lopes da Silva, 2005). However, we argue that interpretations of EEG rhythms in terms of mechanisms, state dependence, and functional correlates should take into account their partially overlapping origins.

## Conflict of Interest Statement

The authors declare that the research was conducted in the absence of any commercial or financial relationships that could be construed as a potential conflict of interest.
